# Replacement of the Trabecular Meshwork Cells—A Way Ahead in IOP Control?

**DOI:** 10.3390/biom11091371

**Published:** 2021-09-16

**Authors:** Xiaochen Fan, Emine K. Bilir, Olivia A. Kingston, Rachel A. Oldershaw, Victoria R. Kearns, Colin E. Willoughby, Carl M. Sheridan

**Affiliations:** 1 Department of Eye and Vision Science, Institute of Life Course and Medical Sciences, University of Liverpool, Liverpool L69 3BX, UK; xiaochen.fan@liverpool.ac.uk (X.F.); emine.kubra.bilir@liverpool.ac.uk (E.K.B.); olivia.kingston@liverpool.ac.uk (O.A.K.); vkearns@liverpool.ac.uk (V.R.K.); 2 Department of Musculoskeletal and Ageing Science, Institute of Life Course and Medical Sciences, University of Liverpool, Liverpool L69 3BX, UK; Rachel.oldershaw@liverpool.ac.uk; 3 Genomic Medicine, Biomedical Sciences Research Institute, Ulster University, Coleraine BT52 1SA, UK

**Keywords:** trabecular meshwork, glaucoma, cellular transplantation, stem cells, iPSC, MSCs

## Abstract

Glaucoma is one of the leading causes of vision loss worldwide, characterised with irreversible optic nerve damage and progressive vision loss. Primary open-angle glaucoma (POAG) is a subset of glaucoma, characterised by normal anterior chamber angle and raised intraocular pressure (IOP). Reducing IOP is the main modifiable factor in the treatment of POAG, and the trabecular meshwork (TM) is the primary site of aqueous humour outflow (AH) and the resistance to outflow. The structure and the composition of the TM are key to its function in regulating AH outflow. Dysfunction and loss of the TM cells found in the natural ageing process and more so in POAG can cause abnormal extracellular matrix (ECM) accumulation, increased TM stiffness, and increased IOP. Therefore, repair or regeneration of TM’s structure and function is considered as a potential treatment for POAG. Cell transplantation is an attractive option to repopulate the TM cells in POAG, but to develop a cell replacement approach, various challenges are still to be addressed. The choice of cell replacement covers autologous or allogenic approaches, which led to investigations into TM progenitor cells, induced pluripotent stem cells (iPSCs), and mesenchymal stem cells (MSCs) as potential stem cell source candidates. However, the potential plasticity and the lack of definitive cell markers for the progenitor and the TM cell population compound the biological challenge. Morphological and differential gene expression of TM cells located within different regions of the TM may give rise to different cell replacement or regenerative approaches. As such, this review describes the different approaches taken to date investigating different cell sources and their differing cell isolation and differentiation methodologies. In addition, we highlighted how these approaches were evaluated in different animal and ex vivo model systems and the potential of these methods in future POAG treatment.

## 1. Introduction

Loss of vision due to glaucoma is one of the leading causes of blindness worldwide [1]. Glaucoma is characterised by an irreversible optic neuropathy, and elevated intra ocular pressure (IOP) is a major risk factor; reducing IOP is the primary treatment in the management of glaucoma. Increased outflow resistance in the conventional outflow pathway of the eye occurs at the trabecular meshwork (TM) in primary open-angle glaucoma (POAG) [2,3,4]. The TM is a complex 3D porous tissue that facilitates flow of aqueous humour from the anterior chamber of the eye to Schlemm’s canal [5,6,7]. In POAG, increased resistance to outflow of aqueous humour at this structure coincides with altered extracellular matrix (ECM) production, remodeling and stiffness, and a decrease in its cellularity [8,9]. A potential therapeutic strategy in POAG is repopulation of TM cells, which may compensate for the decreased cellularity in the glaucomatous TM, modify the abnormal ECM, and result in a reduction in IOP [7,10]. The complexity of the existing ECM architecture in both health and disease undoubtedly influences cellular behavior in repair, replacement, and regenerative scenarios. As such, changes in the ECM landscape with age and glaucoma are outlined eloquently in greater detail elsewhere [8,11,12]. The options to replace lost TM include either repair or regeneration of the TM from resident TM progenitor/differentiated cells or the direct transplantation of new rejuvenated cells from various sources, including adult TM, TM progenitors, induced pluripotent stem cells (iPSCs), or mesenchymal stromal cells (MSCs). In recent years, exploration of these approaches has shown promise in restoring normal IOP through repair and regeneration of the TM in animal models, but this is yet to be explored in humans. This review outlines the work to date on these approaches and discusses the progress and the challenges that accompany them. 

## 2. Cell Type

### 2.1. The TM Cell

Transplantation of adult TM cells poses several key challenges. The anterior chamber is immune privileged and, as such, obtaining autologous cells is an attractive option. However, obtaining TM cells for propagation, expansion, and/or modification would require additional invasive surgery. Such surgery itself would compromise the blood–aqueous barrier and create additional immunological issues to a transplantation procedure. In addition, expression of glaucoma related genes can be found in cells throughout the outflow pathway. In particular, genes with known pathological links to POAG, such as myocilin (MYOC) [13], angiopoietin-like 7 (ANGPTL7) [14], and caveolins (CAV1 and CAV2) [15], if carrying mutations would likely require modification prior to transplantation. A further issue is the lack of cell specific markers for isolating and characterising the TM cells. The lack of specific markers throughout the various locations within the TM structure has been a source of frustration in TM research for decades. A recent consensus on markers was established in 2018 [16]. The panel of markers included chitinase-3 like-1 (CHI3L1) [17], matrix GLA protein (MGP) [18], aquaporin-1 [19], alongside glucocorticoid-induced up-regulation of myocilin (MYOC) [16]. However, more recently, Patel et al. (2020) used single cell RNA sequencing (scRNAseq) to identify 12 distinct cell types in the conventional outflow pathway with region-specific expression of certain genes [20]. As expected, the predominant cell type within the outflow pathway was the TM cell, and two specific TM cell expression clusters termed TM1 fibroblast-like and TM2 myofibroblast-like cells were identified [20]. The phenotypic differences in TM cells within the TM has been appreciated for some time, as TM cells in uveal/corneoscleral meshwork display endothelial and macrophage properties, whilst those residing in the JCT region display a fibroblastic spindle shaped morphology. Interestingly a specific macrophage population of cells was also identified throughout the TM structure. Differing functions and roles have been attributed to the cells populating the TM with uveoscleral TM cells maintaining open flow passageways by secreting anti-thrombotics, engulfing cellular debris, and nullifying reactive oxygen species [20,21,22,23]. JCT TM cells, with their smooth, muscle-like properties, have a role in the generation of outflow resistance [24]. Cells that populate the TM are derived from the neural crest that can display different morphologies depending upon their tissue location. In the context of TM replacement, it therefore creates the question: does a specific TM cell need to be utilised for replacement strategies, or does the transplant environment allow for sufficient cellular plasticity? The merits and the methodologies of harvesting each potential cell type are discussed below. 

### 2.2. The TM Progenitor Cell

(i) Utilising resident stem cells to repopulate the adult population of cells is an attractive option, as it is a process that is prevalent throughout the body, including the eye (e.g., limbus of the cornea epithelium). However, as with other structures in the eye (such as corneal endothelium), this process is thought to not occur (or to happen only in a very limited manner) in the human TM in situ. In 1982, Raviola et al. first identified a new cell type in Schwalbe’s line. This fourth region of the TM (also known as the insert or the transition zone) does not filter aqueous humour, and evidence suggests that stem cells or stem-like progenitor cells reside in this area and that these cells are able to replace TM cells when damage occurs [7,25,26,27]. Cells found in this area have different ultrastructure compared to surrounding TM cells, such as a prominent Golgi apparatus and secretory granules [28]. In addition, Acott et al. reported that surgical insult caused by laser trabeculoplasty initiated cell division by a population of cells in the Schwalbe’s line region to migrate and repopulate the injured sites [29]. Further studies by McGowan et al. found that specific stem cell markers, such as OCT3/4, Wnt-1, PAX6, and SOX2, are expressed in the transition zone between the TM and the corneal endothelium, suggesting that a stem-like cell population may have the potential to repopulate either the TM or the corneal endothelial cells [26]. As a stem-like cell population serves to be on standby to repopulate the TM when damage occurs, the reason why these cells do not repopulate the TM in age and glaucoma when TM cell numbers decrease remains unknown [17,27,30]. It is not unreasonable to hypothesise that changes in genetic, structural, and mechanical properties in the TM occurring as a result of glaucoma may be preventing these progenitor cells from differentiating to replace lost TM cells or actually become lost themselves. Indeed, recently, Sundaresen and colleagues observed that there were reduced numbers of cells with specific stem cell markers ABCG2, neural crest marker p75, and AnkG in older patients compared to younger patients and that the reduced stem/progenitor cell numbers corelated with a reduction in TM tissue cellularity overall [31]. To say that a singular stem cell population exists in one area of the TM may be over simplistic. Not only does it overlook cellular plasticity, but it would also differ from elsewhere in the body. Stem cell populations are largely heterogeneous compared to other cell subpopulations [32]. This can make it difficult to fully understand their physiology and predict their path of differentiation, hindering their use in studies that rely on differentiation into specific cell types due to low efficiency [33]. Heterogeneity of cell types is influenced by tissue physiology and pathology [34] and, thus, within a small area, different sub-populations of stem cells can be present at one time due to differing external microenvironments. In a tissue like the TM, for example, cells are exposed to variations in pressure throughout the day which may not be uniform across the tissue due to the segmental nature of aqueous humour outflow [8,35]. The TM also has distinct regions with different properties such as differing extracellular matrix components, structure, and flexibility [36], which also influences how cells respond to specific growth factors such as transforming growth factor beta (TGFβ) (see also [8,9,37,38,39,40,41]). Therefore, the expression profile of cells in different areas of the TM at any given time are most likely very different and require further understanding of the distinct sub populations of cells within the tissue. This challenge is being addressed with differential harvesting of the sub-populations of SC from TM (outlined in below section about isolation and expansion of TM progenitor cells). In addition, scRNAseq technologies are being utilised to identify heterogeneous sub-populations and observe developmental trajectories of individual stem cells in healthy development and disease [32,42]. The ability to map certain genes to sub-populations of stem cells facilitates tracking the trajectory of which stem cell populations differentiate into terminally differentiated cell types within a tissue [32]. This may be crucial in understanding (and thus making use of potential clinical benefits) as well as identifying disease-associated genes within specific stem cell sub-populations [32].
(ii) Isolation and Expansion of TM Progenitor Cells

Further study of the progenitor population resulted in a plethora of approaches to isolate and propagate them. For brevity and relevance, we focused solely on those methods adapted towards human tissue.

Adherent Culture: Nadri et al. dissected TM tissue from human corneal rims. The TM tissues were then incubated with collagenase for 1 h. The isolated stroma tissue segments were further cultured in low glucose DMEM supplemented with 20% serum and basic-FGF for isolating the TM progenitor cells (Table 1). The TM progenitor cells were then expanded in the same medium by two passages. Nadri et al. reported that these cells expressed mesenchymal stroma cell markers and had the ability to form spheres and were differentiated into osteocytes and adipocytes [43]. This study showed that basic FGF and 20% FBS facilitated sub-culture of the stem progenitor cells.

(a) Side population cell sorting

Du et al. expanded TM progenitor cells by culturing human TM cells in a defined specific stem cell growth medium (SCGM) containing multipurpose reduced serum (see Table 1) and isolated TM progenitor cells from TM cell population by sorting side-populations (SP) using fluorescence-activated cell sorting (FACS) [55]. Whilst SP analysis was shown to be a useful method for identifying stem cell populations in other tissues, especially in the absence of specific cell surface markers, the toxicity of DNA-binding Hoechst can limit cell survival after SP analysis and cell sorting [56]. Moreover, it was shown that SP analysis can miss neural progenitor cells from sphere cultures [48].

(b) Neurosphere assay

As the TM outflow pathway is derived from the neural ectoderm, the neural sphere assay was used to obtain progenitor enriched cultures [49,57,58]. Sphere formation assays allow a small population of cells to form neurospheres in non-adherent serum-free medium conditions supplied with epidermal growth factor (EGF) [59]. Neurospheres were shown to enrich progenitor cell populations (such as transit amplifying cells) [60,61] but not quiescent stem cells. Different research groups [57,58]) utilised sphere forming assays from TM cells. Gonzalez et al. isolated TM cells from TM tissue explants as previously described [62] and generated free-floating TM spheres by culturing the TM cells in StemSpan™ Serum-Free Expansion Medium (SFEM) (see Table 1). The TM spheres were expanded in SFEM for 3 months [49]. Microarray and PCR results showed differentially expressed genes between TM spheres and primary TM cells. The TM spheres were also found to express leukaemia inhibitory factor (LIF), which played a critical role in maintaining the undifferentiated state of neural stem cells [63], and nestin (NES), which was a marker for neural progenitor cells [64]. When the TM spheres were incubated with the serum, they differentiated into monolayer cells with indistinguishable morphology and similar gene expression to TM cells. This proved that TM progenitor cells could be successfully isolated by the sphere forming assay and had the ability to differentiate into TM cells. Zhang et al. introduced a novel 3D aspect to the cell culture conditions by embedding isolated cultured TM cells in thick (3D) Matrigel [50], which subsequently formed spheres after 48 h [65]. They reported that 3D Matrigel could maintain the TM phenotype and the progenitor status of the TM progenitor cells [65]. They showed that the TM progenitor cells cultured on thin (2D) Matrigel [50] continually lost this phenotype. TM progenitor cells cultured in 3D Matrigel expressed higher TM cell markers (such as AQP1, CHI3L1, MGP, and AnkG) and embryonic stem cell and neural crest cell markers (such as KLF4, NANOG, OCT4, SOX2, and PDGFRβ) than the cells cultured on 2D Matrigel. The 3D stem cell culture method is attractive in that it can simulate normal cell morphology, proliferation, and differentiation more accurately [66] and can upregulate some functional cell markers in comparison with the 2D culture [44].

(iii) Identification of TM progenitor cells

Although TM progenitor cells have attracted attention as a potential way to treat POAG, the biological properties and the specific markers of this new cell type are still elusive. Previous studies identified a group of genes which are highly expressed in TM progenitor cells (Table 2). These studies showed differentially expressed genes between TM progenitor cells and TM cells. A few studies also highlighted a group of stem cell-related genes shown to be expressed in the TM tissue and/or the transition zone between the TM tissue and the corneal endothelium. It is likely there is associated heterogeneity in stem cell-related gene expression, and those findings could help elucidate the location of the TM progenitor cells and the migration of the TM progenitor cells during wound healing (Table 3). 

### 2.3. Induced Pluripotent Stem Cells Derived TM cells (IPSC-TM Cells)

Non-ocular cell sources for TM replacement have focused on induced pluripotent stem cells (iPSCs). Initial studies producing TM-like cells (differentiated TM) used mouse fibroblast induced iPSCs in a co-culture method with immortalised TM cells for up to 21 days [68]. Subsequently, human iPSCs were differentiated into TM-like cells by culturing iPSCs with TM cell-derived ECM in a specially designed TM growth medium containing conditioned media for 30 days [69]. Analysing cytokines of the medium conditioned by HTM cells, Wang et al. generated a differentiation protocol to differentiate iPSCs into TM-like cells. The iPSCs derived from renal urethra epithelial cells were exposed to the recombinant cytokines that bind to receptors, including TGFβ1, nerve growth factor beta, erythropoietin, prostaglandin F2 alpha, and epidermal growth factor [70]. A recent novel approach to differentiate human iPSCs from human dermal fibroblasts into TM cells via an intermediate neural crest cell (NCC) stage using conditioned media and ECM from primary human TM cells was reported [46,71]. TM-like cell populations in these studies were similar to normal human TM cells morphologically, expressed characteristic markers, and displayed phagocytic capability, indicating that these TM-like cells might function in vivo to modulate outflow resistance [46,68,69,71].

Interestingly, the origin of the iPSC cells influences the transcriptional profile of differentiated cells. Zhu et al. demonstrated with the principal component analysis of RNA that iPSC-TM was distinct from both the originating fibroblasts and the keratinocytes, with the resultant iPSC relatively tightly clustered to primary TM (pTM) form. However, when iPSC-TM were derived from different donors, results showed that donors tended to cluster with one another in the same donor samples. This indicated that the genetic background of the donor affects the transcriptional profile of TM cells and that individual differences are maintained by iPSC-TM [72]. Compared to dermal fibroblast cells from the skin, renal urethra epithelial cell-originated iPSCs were found easier to collect from urine and thus potentially more clinically translatable (Table 4) [70].

### 2.4. Mesenchymal Stem Cell Derived TM Cells (MSC-TM Cells)

Mesenchymal stem cells (MSCs) were widely researched as new therapeutic agents in the cell-based therapy of glaucoma [75,76]. Morgan et al. showed that human MSCs have many important similarities with human TM cells. Human TM cells exhibit surface markers including CD73, CD90, CD105, and CD146 expression consistent with the human MSCs. Moreover, the expression level of transcriptional factors responsible for cell potency and proliferation (SOX2, POU5F1 and NOTCH1) was similar in TM cells as in MSCs [77]. Contrarily, expressions of five TM markers, tPA, LDL receptor, CHI3L1, MGP, and MYOC, were designated to distinguish between TM cells and MSCs [78].

The investigation of potential differentiation of MSC into TM cells started with Manuguerra-Gagné et al. coculturing florescent dye tracked mouse bone marrow derived-MSCs together with mouse TM cells in vitro. After 7 days of co-incubation, the MSC population that directly contacted TM cells failed to acquire the expression of aquaporin and laminin [79]. Differentiation capability of adipose derived mesenchymal stem cells (ADSCs) into corneal keratinocytes sparked interest in ADSC differentiation into TM cells, as TM cells share the same origin as corneal keratinocytes, the neural crest [80]. Zhou et al. demonstrated that human ADSCs can differentiate into TM-like cells using three different methods, including ADSC coculture with TM cells (coculture), exposure of ADSC cells to ECM and conditioned media (CM) from TM cells (ECM + CM), and exposure of ADSC cells to ECM from TM cells and culture in Advanced Minimum Essential Medium (ECM + AdvM). ADSC by co-culturing with TM cells or by culturing on TM ECM + CM generated TM-like cells, as evaluated by phagocytosis and dexamethasone-induced expression of MYOC and CLAN formation. These ADSC-TM cells showed phenotypic similarities to native TM cells, including reduced expression of OCT4 and increased expression of CHI3L1 and AQP1. On the contrary, ADSC-TM cells induced by ECM + AdvM had fewer dexamethasone-induced CLAN-forming cells [81].

## 3. Transplantation Studies

As the understanding of TM cell biology requirements for transplantation or regeneration expands, the use of models is key to advancing these potential technologies for treatment. As with all models, none perfectly recapitulate the in vivo TM, but each has justifiable merits alongside weaknesses to expedite progress in the field. We summarise the experimental work to date for each cell type investigated in various in vivo and ex vivo models (Table 5).

### 3.1. Adult TM Cell Transplantation

As far as we are aware, currently, there are no studies that transplant adult TM cells into animal model systems. However, interestingly, several studies that transplant TM derived stem/progenitor cells into animal models often use another cell type as a control, such as fibroblasts [44,45]. These fibroblast control cells often cause changes in the TM not seen in “wild type”/untreated controls, such as increased inflammatory markers like CD45 and changes in IOP, potentially influencing the perceived outcome of the experiment [44,45]. Whether transplanted adult primary TM cells would elicit the same response is not known. Additional studies using transplanted adult TM cells compared to transplanted TM derived stem/progenitor cells may yield useful comparison data in future animal experiments. 

### 3.2. TM Progenitor Cell Transplantation

As TM progenitor cells proliferate in vitro and can differentiate into functioning TM cells, direct transplantation of TM progenitors also offers a potential therapy for TM cell loss in POAG. In 2013, Du et al. injected human TM progenitor cells, pre-labelled with fluorescent dye DiO, into the anterior chamber of C57BL/6 wild type mice. The TM progenitor cells localised to the murine TM tissue and iris after 1 week, 4 weeks, and 4 months. Up to 4 months after injection, differentiated TM markers were expressed in mice TM tissue, identified by staining the TM with human reactive antibodies. These findings suggested that human TM progenitor cells could be home to the murine TM tissue after anterior chamber injection and differentiation in situ. Furthermore, TM cells derived from TM progenitor cells locate to the TM more so than fibroblasts [44,45]. As TM progenitor cell injection did not cause changes of mice cornea transparency, inflammation, or elevated IOP [44], this boded well for future studies. Following this work, TM progenitor cell transplantation was performed in laser-induced damaged TM [45] and more recently in a POAG mouse transgenic myocilin Y437H mutant model [82]. In 2018, Yun et al. transplanted human TM progenitor cells into laser-damaged mice trabecular meshwork by injection. The human TM progenitor cells were found to be home to the injured TM region, which reconstructed (in part) the damaged trabecular meshwork structure and maintained a normal IOP and outflow facility [45]. Whilst this evidence suggests that the TM cells preference was to locate to the TM region, it was noted that the TM progenitor cells were also found in the other eye regions such as the iris. Since the progenitor cells may differentiate into undesired phenotypes in the eye, this would obviously be problematic. The injury model, the passive delivery, the AH outflow dynamics, and the injection delivery method are all likely to impact the final location of the injected progenitor cells. More recently, Xiong et al. transplanted these TM progenitor cells into the Tg-Myoc^Y437H^ mice and showed that TM progenitor cells differentiated into TM cells, remodeled the extracellular matrix, and decreased the IOP in the Tg-Myoc^Y437H^ mice after transplantation.

### 3.3. iPS Cell Transplantation

Studies involving the transplantation of iPSC-TM are still in their infancy compared to elsewhere in the eye [85]. However, the progress of the studies offers a potential therapy for TM replacement in POAG. Zhu et al. used a glaucoma mouse model that constitutively expresses a transgene containing human myocilin with a pathogenic Y437H mutation (Tg-MYOC^Y437H^) and displays multiple glaucomatous phenotypes, including TM cell loss, elevated IOP, and progressive RGC loss. Beginning at the age of 4 months old, Tg-MYOC^Y437H^ mice exhibit the first signs of TM cell loss and start to develop elevated IOP by 6 months old compared to age-matched wild type mice. [74]. Mouse iPSCs were differentiated toward a TM cell phenotype (iPSC-TM) and injected into the anterior chamber of Tg-MYOC^Y437H^ mice representing both early [74] and late [73] stages of the disease. Twelve weeks after transplantation, both young (4-month-old) and old (6-month-old) models demonstrated increased numbers of TM cells, restored aqueous humour outflow, lowered IOP, and preservation of retinal ganglion cells (in younger eyes only) compared to controls. Although some implanted iPSC-TM cells were observed within the TM, the increase in TM cell number was in part due to the proliferation of resident TM cells. Whilst the better outcomes were observed in the younger eyes, it was unlikely due to fewer TM cells lost in those eyes prior to transplant but rather due to a stronger repair process [73]. Other variables in younger eyes include structural or physical changes to the TM, such as stiffening, reduction in autophagy, reduced matrix turnover, or phagocytosis by TM cells [73,86]. The influence of immune response was addressed in the iPSC studies by utilising iPSC-TM cells derived from mice with the same strain and genetic background, including MHC self-antigens, as the recipient animals. Therefore, the influence of immune rejection was significantly diminished in this study, as evidenced by the lack of increased infiltration of leukocytes of macrophages to the anterior chamber of the eye as well as the TM [74].

In addition to animal transplantation studies, iPSC-TM generated from human iPSCs were transplanted into the TM of human eyes obtained from donors by utilising a perfused organ culture system [69,72] (Abu-Hassan et al., 2015; Zhu et al., 2020). Zhu et al. observed the resident TM cell proliferation up to 7 days post-transplantation of TM-like iPSCs. No further increase in the resident TM was observed in the following week, and there was no change in outflow facility observed in iPSC-TM transplanted eyes after 14 days [72]. In addition, TM-like iPSCs were transplanted into human anterior segments that were treated with saponin to model cell death in glaucoma [69]. In examining the IOP response to cell transplantation, only differentiated TM-like iPSCs were able to restore the IOP homeostatic response in anterior segments compared to control cell transplantations of dermal fibroblasts, embryoid bodies, human umbilical vein endothelial cells, and mock transplantation (no cells added) [69]. The shorter observational time points (2 weeks) in the perfused organ culture system due to tissue degradation was insufficient to observe the slower changes that occurred in mouse eye models (6–9 months). 

### 3.4. MSCs Transplantation

Initial transplantation studies of MSCs used bone marrow derived MSCs (BM-MSC) from C57BL/6 mice that were injected into the anterior chamber of the laser-induced rat glaucoma model [79]. Following injection of MSCs a reduction in IOP occurred, coinciding with induced TM regeneration (proliferation). This was not observed in eyes receiving injections of control cells, lymphocytes or CD45 positive cells. Interestingly, the BM-MSCs remained in the anterior chamber transiently (none detected at 96 h post injection) and did not induce an inflammatory response or integrate within the TM itself. However, nestin positive cells (potential neural progenitor), which are normally only seen in the murine ciliary body epithelium, were detected in the TM. Additional studies in a rat ocular hypertension (OHT) model in which post-episcleral vein cauterization induced a decrease in AH outflow and consequently an increase in IOP were also utilised. Similarly, a reduction in IOP was observed following BM-MSC transplantation in this rat OHT model for up to 14 days, and injected BM-MSCs were located near the iridocorneal angle, on the iris, on the corneal endothelium, in ciliary processes, and in the TM [83]. MSCs have also been utilised from sources other than bone marrow. Indeed, adipose derived MSC (ADSC) were also investigated, and, following intracameral transplantation into adult C57BL/6 wild type mice, both ADSCs and ADSC-TM cells located and integrated into the TM tissue compared to control fibroblasts cells, which scattered throughout the anterior chamber. The effect was more so with ADSC-TM; there was no observed IOP elevation in either ADSC or ADSC-TM cell injected eyes, whereas fibroblast-injected eyes demonstrated an elevated IOP for up to 1 month [81]. To date, no transplantation of MSCs into larger-eyed (pig, bovine, primate, or human perfused anterior segments) has been studied. However, there was one small, limited study in which human cord blood-derived stem cells (HUBC)s injected into TM damaged rabbit eyes showed preservation of TM structure and endothelial cellularity compared to damaged, non-injected controls [84]. 

The mechanism by which transplanted cells preferentially locate to the compromised TM structure is vitally important. ADSC and ADSC-TM are more likely to locate to the TM and are less likely to adhere to the iris and the CE than fibroblasts [81]. The preferred homing of ADSC and ADSC-TM cells may in part be mediated through the SDF–CXR4 axis, which is a key pathway in endothelial cell migration and repair in the eye [87] (see complementary review in this journal by Mallik et al. 2021). Strategies to facilitate delivery of cells to the TM include biological manipulations (such as SDF) but also other delivery technologies. One such approach was successfully developed for MSC using magnetic nanoparticles to deliver the cells into the TM [78]. ADSCs labelled with Prussian blue nanocubes with superparamagnetic iron oxide nanoparticles were steered to the TM tissue in organ cultured porcine eyes using a magnetic field. In total, 50% to 75% of injected MSCs were delivered to TM tissue efficiently, and no significant differences were observed between the duration of magnet exposure [78].

## 4. Conclusions

Recent studies made tremendous progress in demonstrating the potential effectiveness of regenerative therapies using iPSCs, TM progenitor cells, and MSCs in restoring TM tissue and reducing IOP. Whilst this is promising, the understanding of whether these cells restore the function of the TM long-term, how they aid in IOP regulation, and whether they would be effective in human glaucoma patients are still unknown. Understanding how these cells function in glaucomatous environments will be key if the progression of these approaches into the treatment of patients is to be realised. Ex vivo and artificial models that provide a similar environment to glaucomatous TM in humans may be useful tools in exploring these approaches in the future before patient treatment can be realised.

## Figures and Tables

**Table 1:** Methods of isolation and expansion of trabecular meshwork progenitor cells.

Ref	Method of TM Progenitor Cell Isolation	TM Progenitor Cell Expansion Medium	Similar Methods
[42]	Cells were isolated from the TM tissue and cultured in specific culture medium on adherent culture conditions for sorting the TM progenitor cells	Low glucose DMEM supplemented with 20% serum and 200 ng/mL basic-FGF	N/A
[43]	Cells were isolated from the TM tissue. TM progenitor cells were sorted from TM cells by SP assay and cultured in stem cell medium on adherent culture conditions	Stem cell growth medium (SCGM) containing multipurpose reduced-serum media (Opti-MEM) supplemented with 5% fetal bovine serum (FBS), 10 ng/mL epidermal growth factor (EGF), 100 μg/mL bovine pituitary extract, 20 μg/mL ascorbic acid, 200 μg/mL calcium chloride, 0.08% chondroitin sulfate, 100 IU/mL penicillin, 100 μg/mL streptomycin, and 50 μg/mL gentamicin	[44,45,46,47]
[48]	Cells isolated from TM tissue were cultured in stem cell culture medium in suspension. TM progenitor cells were isolated by sphere formation assay	StemSpan™ Serum-Free Expansion Medium (SFEM) (StemCell Technologies, Seattle, WA, USA)	N/A
[49]	Cells were isolated from the TM tissue and cultured in neural stem cell culture medium in suspension. TM progenitor cells were isolated by sphere formation assay	DMEM (low glucose), containing 20 ng/mL EGF (Peprotech, UK), 20 ng/mL b-FGF (Peprotech, UK), 5 ug/mL Heparin Sodium (Sigma-Aldrich, UK) and 1X B27 supplement (Thermo Fisher Scientific, UK) at 37 °C, 5% CO2	[26]
[50]	Cells were isolated from the TM tissue and cultured in 3D Matrigel with stem cell culture medium. TM progenitor cells were isolated by sphere formation assay	3D Matrigel made by adding 50% diluted Matrigel in MESCM + 5% FBS	N/A
[51]	Cells were isolated from the TM tissue and cultured in specific culture medium on adherent culture conditions for sorting the TM progenitor cells	DMEM (low glucose), containing 10% FBS, 4 mM L-GlutaMAX™, 1 mM sodium pyruvate, 1% nonessential amino acids, and 1% penicillin–streptomycin, on uncoated tissue culture plastic.	[52]
[53]	Cells isolated from the transition zone between TM and cornea endothelium (the tissue without pigmented TM and translucent peripheral endothelium) were cultured in the specific culture medium in 2D Matrigel	OptiMEM1 with recombinant human epidermal growth factor (h-EGF, 10 ng/mL), recombinant human basic fibroblast growth factor (h-bFGF, 20 ng/mL), bovine pituitary extract (100 μg/mL), L-ascorbate (20 μg/mL), chondroitin sulfate (0.08%), calcium chloride (0.9 mM), and knockout serum replacement (5%).	[54]

**Table 2:** Identification and detection methods of specific TM progenitor markers in vitro under different progenitor cell culture conditions.

Ref	TM Progenitor Cell Culture Conditions	Detection Method	TM Progenitor Cell Markers
[42]	Adherent culture conditions	Flow cytometric analysis	CD105, CD90, CD44, and CD166
[43]	Adherent culture conditions	PCR and/or immunofluorescence	ABCG2, NOTCH-1, MUC1, and ANKG
[47]	Adherent culture conditions	PCR, western blotting, immunofluorescence, and/or flow cytometric analysis	NES, OCT3/4, α5 integrin, and α5β1 integrin
[48]	Suspension conditions (sphere formation)	Microarray and PCR	NES, LIF, BDNF, IL6, and CSF3
[50]	Suspension conditions (sphere formation in 3D Matrigel)	PCR and/or immunofluorescence	OCT4, SOX2, KLF4, Vim, AQP1, CHI3L1, MGP, and AnkG
[51]	Adherent culture conditions	Flow cytometric analysis and immunocytochemistry	CD73, CD90, and CD105
[54]	Adherent culture conditions	RCR and/or immunofluorescence	ZO-1, Na+/K+ ATPase, PITX2, and SOX10

**Table 3:** Detection of progenitor cell markers in human TM tissue and the method of detection. TZ: transition zone between TM and cornea endothelium; TM: trabecular meshwork; SC: Schlemm’s Canal endothelium; JCT: TM juxtacanalicular region; PE: peripheral endothelium; N/A: not applicable.

Ref	Detection Methods	Markers Expressed in the TM Region	Markers Expressed in the TZ Region	Markers Expressed in the SC and JCT Region	Markers Expressed in the PE Region
[25]	IHC (fluorescence, cryosections)	Unwounded corneas: nestin and telomerase. Post-wounded corneas: SOX2, OCT3/4, Wnt-1, and PAX6	Unwounded corneas: nestin, alkaline phosphatase, and telomerase. Post-wounded corneas: SOX2, Wnt-1, PAX6, and OCT3/4	Unwounded corneas: telomerase. Post-wounded corneas: PAX6 and Wnt-1	Unwounded corneas: nestin and telomerase. Post-wounded corneas: SOX2, OCT3/4, and Wnt-1
[30]	IHC (fluorescence, Paraffin sections)	N/A	P75 and ABCG2	N/A	N/A
[53]	IHC (fluorescence, cryosections)	Vimentin and CD44	Nestin, Vimentin, Lgr5, SOX2, CD34, HNK1, Prdx6, Pitx2, Lgr5, TERT, P75, and CD44	N/A	Lgr5, Prdx6, nestin, CD34, and TERT
[67]	RNAscope multiplex fluorescent assay	PDPN, DES	PDPN	PDPN, CHI3L1, PECAM, POSTN, TFF3, DES, Pecam1, and POSTN	AOP1

**Table 4:** Methods of iPSC differentiation into TM cells and TM cell-specific markers and detection methods.

Ref	Model	Differentiation Method	Detection Method		TM Markers
[46]	Human		Two step induction (first step NC, then TM-ECM) *(10–14 days)*	RT-PCR, immunohistochemistry (IHC)	MYOC, CHI3L1, NGFR, HNK1, ANGPTL7
				
[68]	Mouse and Human		Co-culturing with TC inserts *(21 days)*	IHC, phagocytosis, proteomics	COL4A5, MGP, MYOC, TIMP3, MMP3
[69]	Human and Porcine		Generating EBs and TM-ECM *(30 days)*	POC, IOP measurement, IHC, phagocytosis, qPCR, WB	AQP1, CHI3L1, WNT1, α3 integrin
[70]	Human		Feeder free differentiation *(7 days stage 1 and 14 days stage 2)*	RT-PCR, immunocytochemistry (ICC), mRNA sequencing	LAMA4, TIMP3, AQP1, COL4, MYOC
[71]	Human and Porcine	Two step induction (first step NC, then TM-ECM) *(10–14 days)*	3D culture, SEM, ICC, western blot (WB), IOP measurement		MYOC, αSMA, Fibronectin, COL4
[72]	Human	Co-culturing with TC inserts *(30 days)*	IOP measurement, qPCR, IHC, mRNA sequencing		Vimentin, AQP1, MGP, COL4, MYOC, COL1, TIMP3
[73]	Mouse	Conditioned TM media *(14 days)*	TEM, WB, IHC, IOP measurement		MYOC, Calnexin
[74]	Mouse	Conditioned TM media *(14 days)*	POC, IOP measurement, IHC		LAMA4, TIMP3, MYOC, COL4

**Table 5:** A summary of studies to date that transplanted iPSC, MSC, or TM progenitor cells into animal and organ culture models. Outputs are summarised to reflect the characterisation of cells in situ following transplant, their location, inflammation in comparison to control cells, and their effect on IOP. IF-immunofluorescent staining.

Ref	Model	Cell Type	Output
***TM Progenitor Cells***			
[44]	(C57BL/6) Wild-type mouse	DiO-Labelled trabecular meshwork stem cells (TMSCs)	**TM Markers:** CHI3L1 and MUC1 positive (after 1 week). **Found:** primarily localised to TM and few cells in the iris (up to 4 months). **Control cells:** fibroblast (CHI3L1 or MUC1) found mainly corneal endothelium and lens epithelium. **Additional effects:** TMSCs or fibroblasts had no effect on corneal transparency, corneal endothelial morphology, or IOP. Only fibroblast-injected eyes displayed an inflammatory response, with increased CD45 in the TM.
[45]	(C57BL/6) Mice with laser-photocoagulation of the TM	DiO-labelled human TMSCs	**TM Markers:** AQP1 and CHI3L1 detected by IF staining. **Found:** Mostly at laser treated sites in the TM after 2 weeks and to a lesser extent throughout the TM. **Control cells**: Fibroblast cells—detected in the iris and to a lesser extent throughout the TM. **Additional effects:** Structure of laser-damaged TM was restored after 4 weeks only in TMSC-injected mice. TMSC-injected mice also had reduced CD45 and SPARC expression compared to damaged, non-injected controls.
[82]	Tg-MYOC^Y437H^ POAG mice	DiO-labelled human TMSCs	**TM markers:** AQP1 and CHI3L1 positive (after 2 months). **Found:** The DiO-labelled TMSCs were detected at the TM region (up to 2 months). **Control:** Age-matched wild-type (WT) mice. **Additional effect:** The **IOP** of the Tg-MYOC^Y437H^ mice was decreased after one month of TMSC transplantation; TMSCs remodelled the **extracellular matrix** in the Tg-MYOC^Y437H^ mice.
***Mesenchymal Stem Cells from Other Tissues***			
[78]	Organ cultured porcine eyes	Human adipose derived MSCs labelled with magnetic Prussian blue nanocubes (PBNC-MSCs)	**TM Markers:** Not investigated. **Found:** PBNC-MSCs were detected at significantly higher levels in the TM than unlabelled MSCs after 15 min of exposure to magnets to steer the direction of cells. **Control cells:** Unlabelled MSCs. **Additional effect:** Labelled cells evenly distributed around the entire TM circumference after magnet exposure. PBNCs had no adverse effect on MSC viability or multipotency (when differentiating into adipogenic and osteogenic phenotype) in vitro, however, this was not measured in vivo.
[79]	Rat glaucoma model (laser induced IOP elevation)	Mouse bone marrow MSCs	**TM Markers:** MSCs were not found to differentiate—IF staining was negative for AQP1, FN, LM, and PAX6. **Found:** At the laser damaged site between 24 and 48 h. **Control:** Laser damaged eyes without MSC injection. **Additional effects:** BM-MSCs injection caused more efficient reduction of IOP. Eyes injected with MSCs did not present with scarring after 1 month. Conditioned medium from MSC cell culture caused reduced IOP suggesting change due to paracrine secretions of MSC cells. MSCs and conditioned medium also induced proliferation of progenitor cells located at ciliary body.
[81]	(C57BL/6) Wild-type mouse	Human DiO-labelled adipose derived stem cells (ADSCs) and ADSC-derived-TM cells (ADSC-TM)	**TM markers:** CHI3L1 and AQP1. **Found:** ADSC and ADSC-TM integrated throughout all layers of TM tissue (determined by DiO labelling and IF staining) 30 days after injection to the anterior chamber. **Control:** Primary ADSCs and fibroblasts **Additional effect: IOP** and **outflow facility** were maintained in mice injected with ADSC and ADSC-TM but not in fibroblast control.
[83]	Rat ocular hypertension model	Rat Q-Dot labelled bone marrow MSCs	**TM markers:** COL3, COL4, α-SMA. **Found:** 24 days after the injection, MSCs were found located near the iridocorneal angle, on the corneal endothelium, and in the TM. **Control:** Hypertensive eyes injected with media, hypertensive eyes injected MSCs suspension, hypertensive eyes injected with differentiated MSCs suspension, and normotensive eyes injected with MSC suspension. **Additional effect:** Only the MSCs group decreased the IOP significantly compared with the other groups for 13 days.
[84]	Rabbit with laser diode treatment	Human cord blood-derived stem cells (HUCB)	**TM Markers:** Not investigated. **Found:** HUCB cells lined trabecular beams confirmed by presence of markers CD34/CD44, and PKH26 labelled cells. **Control:** Laser-damaged eyes without HUCB injection. **Additional effects:** HUBC-injected eyes had preserved TM structure and endothelial cellularity compared to damaged, non-injected controls. Neither group showed significantly different IOP to baseline after laser treatment.
***iPSCs***			
[69]	Organ cultured human eyes	Human QDot labelled TM-like iPSCs	**TM Markers:** AQP1, Chi3L1, WNT, α3 Integrin. **Found:** TM-like iPSCs integrated into the TM. **Control:** QDot labelled TM cells, dermal fibroblasts, embryoid bodies, and HUVEC cells. **Additional effect:** IOP homeostatic response to 2x pressure challenge was restored by transplanting TM cells/TM-like iPSCs.
[72]	Adult human donor eyes in perfused organ culture	Human GFP labelled iPSC-TM	**TM Markers:** MGP, MYOC, Vimentin, AQP1, COL4, and COL1. **Found:** GFP labelled iPSC-TM cells were not detectable in recipient eyes. **Control:** The second eye from the same donor was maintained as a non-injected control. **Additional effect:** Transplantation of iPSC-TM stimulates proliferation of the recipients’ endogenous TM cells in perfusion cultured human eyes from aged donors. In total, 71.4% of iPSC-TM recipients’ eye displayed normal outflow facility for two weeks after transplantation.
[73]	Young transgenic mice expressing form of human myocilin (Tg-MYOC^Y437H^)	Mouse iPSC-TM	**TM Markers:** MYOC and calnexin. **Found:** IOP was lower and outflow facility was improved compared to untreated controls (12 weeks). **Control:** Wild type mice and PBS injected Tg-MYOC^Y437H^ mice were used as the control group. **Additional effect**: The number of endogenous TM cells increased.
[74]	Young transgenic mice expressing a pathogenic form of human myocilin (Tg-MYOC^Y437H^)	Mouse iPSC-TM	**TM Markers:** MYOC, TIMP3, LAMA4. **Found:** Injected iPSC-TM cells integrated into the TM (12 weeks). Injected cells were detected also in the endothelial cell layer of the Schlemm’s canal, the corneal epithelium stroma, and the area surrounding the injection site (9 weeks). **Control:** Fibroblasts injected transgenic mice, PBS injected transgenic mice, and wild type mice were used as control groups. **Additional effect:** IOP was significantly reduced in iPSC-TM–treated mice. The cellular density of the TM in transgenic iPSC-TM recipient mice was significantly higher than PBS injected transgenic controls and similar to WT animals.

## Data Availability

Not applicable.
